# Reviving public provisioning in US health care

**DOI:** 10.1093/haschl/qxaf013

**Published:** 2025-03-17

**Authors:** Hayden Rooke-Ley, Dana Brown, Colleen Grogan

**Affiliations:** Center for Advancing Health Policy through Research, Brown University School of Public Health, Providence, Rhode Island, 02903; The Democracy Collaborative Foundation, Inc., Shaker Heights, Ohio, 44120; Crown Family School of Social Work, Policy, and Practice, University of Chicago, Chicago, Illinois, 60637

**Keywords:** consolidation, health equity, private equity, public financing

## Abstract

As new approaches of political economy gain ground in some sectors, American health care still reflects many aspects of neoliberalism. In this piece, we build on proposals to reorient health care policy around a new industrial policy for health. A core component of this strategy—and our focus here—is a revival of public provisioning of medical services and pharmaceuticals. Although less prevalent today, forms of public provisioning still exist in vital ways. These models demonstrate how public provisioning can not only address urgent capacity needs—it can promote local ownership, operate as a competitive public option that bolsters worker power, and assure societal return on public investments.

## Introduction

Despite experimentation in certain sectors with new paradigms of political economy, American health care still exhibits the many hallmarks of neoliberalism. The privatization of Medicare typifies an overreliance on market making and consumerism,^1^ even as it drives up costs and builds private power with public dollars.^2^ Widespread corporate consolidation, pursued in the name of efficiency, increases costs and often reduces access for low-income patients. The financialization of care delivery—instantiated by private equity, insurance conglomerates, and profit-oriented “nonprofit” hospital systems—prioritizes returns for investors and corporate management over the production of health. The broader picture is one of neoliberal “re-regulation”: it is not that the health care state has receded—indeed quite the opposite—but that it has been repurposed to empower large corporations and financial investors over popular democratic demands.^3^

In response, calls are emerging for a new health care paradigm to join the existing ferment of industrial policy.^4^ As we have argued elsewhere, this agenda would combat corporate consolidation and empower clinicians, patients, and the health care workforce. This approach would emphasize questions of ownership and governance of health care institutions and would think deliberately about when and where markets should be deployed and how to structure them to foster fair competition. It would also emphasize long-neglected “supply-side” factors: how we build and properly allocate the capacity and labor supply to meet growing care needs.^5^

Core to this agenda should be a revival of public provisioning, which we define as medical care and pharmaceutical production that is owned and governed by public institutions. Public provisioning has always been a feature of American health care delivery, although it began to wane following the Great Society initiatives, which substantially expanded public financing of private infrastructure through public insurance subsidy mechanisms (ie, Medicare and Medicaid). The decline of public provisioning accelerated with the neoliberal turn, as an ideological attraction to privatization and markets took hold.^6^ Nonetheless, as we explain here, public provisioning still exists in vital if less visible ways today. These models demonstrate how public provisioning can promote local ownership, serve as a competitive check on private industry, and assure that society benefits from public health care investments.

## Public provisioning today

Much of public provisioning exists within alternative systems of care that were developed for discrete populations, such as the Veterans Health Administration, the Indian Health Service, and care delivered in departments of corrections. Our emphasis in this piece, however, is to illuminate certain forms of public provisioning that exist within the broad, largely privately administered health care system.

### Public hospitals

Despite only comprising 5% of hospitals, public hospitals provide nearly a third of the nation's level I trauma centers, 45% of the nation's burn care beds, and 27% of pediatric intensive care beds.^7^ Public hospitals are defined as government-owned acute care hospitals and are owned by a state, county, city, or hospital district. Public hospitals are more likely to provide extensive outpatient services, be better integrated with public health departments, and provide culturally and linguistically appropriate care.^8^ Even under highly constrained budgets compared with nonprofit and for-profit hospitals, they can often provide effective, high-quality care.^9-11^

The decline of public hospitals is recent: in 1980, they made up nearly a third of all US hospitals but have since declined by 39%; meanwhile, for-profit hospitals have increased by 32% since 2009.^12^ A key driver here is public-to-private conversions. The evidence suggests privatization (including nonprofit and for-profit) does not increase efficiency but instead undermines access to care. Privatization of hospitals from 2000 to 2018 reduced admissions of low-income Medicaid patients in hospitals that converted to private and also resulted in an aggregate decline in Medicaid patient utilization in markets that experienced privatization.^13^ While federal and state governments substantially expanded Medicaid eligibility from 1983 to 2019, these coverage expansions have been undermined by reductions in accessible capacity. Moreover, public hospitals that privatized to nonprofit and for-profit had higher average mortality rates compared with public hospitals not experiencing privatization during this time period.^13^

Closures have also played a key role in the decline of public hospitals, as local governments are no longer willing to underwrite capital investment and uncompensated care. Yet as private hospitals close, local communities call on the government to respond. For example, HCA Healthcare, a national for-profit chain, announced plans to close Santa Clara's Regional Medical Center's trauma center in 2023 and downgrade labor and delivery, stroke, and heart attack services. Santa Clara County government acquired Regional Medical Center in response, with the help of federal financing from the Federal Emergency Management Agency (FEMA) associated with COVID-19 funding. Similarly, Massachusetts is currently rescuing 5 bankrupt Steward Health Care hospitals—a private equity-backed chain—that could cost taxpayers $700 million by 2027.^14^

These bailouts are part of a larger story of public financing with private control. The growth of taxpayer-financed insurance—from Medicare and Medicaid to tax exemptions for employer insurance—channels public dollars to private hospitals through retrospective compensation for care. Even major capital financing projects, most notably the former Hill–Burton grant program, primarily financed nonprofit private hospital expansion, and Medicare underwrote capital investments funded by tax-exempt bonds. Both of these programs came with few strings attached, such as access to care assurances for all members of society in exchange for this public investment.^15^ As the current hospital landscape suffers from misallocation of capacity—in part due to the financialization of hospitals of all corporate forms^16^—policymakers ought to consider maintaining and upgrading existing public hospitals and a more robust role for new public hospitals through which to direct public capital financing.

### Health and hospital districts

Whereas public hospitals owned by the county have traditionally been located in urban areas, health and hospital “districts” or “authorities” originated to meet care needs in rural areas during the mid-century period of modern capacity expansion. These “special purpose districts” are formed to deliver health services to a defined population and geography. They exist in states across the nation and are especially prevalent in the West: there are 56 hospital districts in Washington State,^17^ 28 in Oregon, and 79 in California.^18^

Oregon's health districts exemplify the range of services and governance structures of health districts. Pursuant to state law, a health district in Oregon can be formed to provide medical and rehabilitative care, health education, and research and promote district residents’ health. The statute grants the local health district the authority to raise revenue through local taxes and to issue bonds, with the voting consent of the individuals in the district. The statute also authorizes the health district to exercise eminent domain. The district may consist of territory in one or more counties and is governed by a board elected by residents in the health districts. Once established, the health district must propose and adopt an annual budget, with a public hearing and opportunity for public input in the ratification process (Oregon Revised Statute 440.360).

In Oregon's coastal Tillamook County, Nehalem Bay Health District is currently building a new 50-bed nursing facility, a community health center, and a pharmacy. It is also standing up housing units specifically to attract health care workers.^19^ To the south, in Lincoln County, Pacific Communities Health District takes a different form: it contracts out operations for its local 25-bed critical access hospital, while its current focus is on developing a seismically resilient water supply source to ensure the local hospital remains functional in the event of an earthquake.^20^ In Northeast Oregon, the Morrow County Health District offers a wide range of services, including a school-based health center, a primary care office, and ambulance services.^21^

As Oregon demonstrates, health districts represent another model of locally governed and publicly accountable care that can adapt to a community's needs. However, raising capital for these local investments is challenging, particularly because these services are often offered in under-resourced rural areas. Investing in these models will likely require cross-subsidization at the state or federal level.

### Public community health centers

Federally qualified health centers (FQHCs), or “health centers,” are federally supported medical clinics that provide care to more than 30 million Americans. While nearly all health centers operate as private nonprofits, a small subset, capped at 5% of the program, are “public entity” health centers (42 U.S.C. § 254b(r)(2)(A)). These health centers are governed by a multi-stakeholder board composed of a majority of patients. Still, operations are tightly interwoven with the public entities affiliated with the health center, such as a county health department, a health or hospital district, or a public academic center. In many instances, the public entity will initiate the application and lead the process alongside the independent board. Once operating, the public entity will oversee and largely control operations. Employees of the health centers, including the physicians, are often indistinct from county employees, with the same compensation structure, retirement benefits, and union representation.

As a result, these health centers become functional extensions of the public entity with which they affiliate. They thus function as a sort of “public–public” partnership for provisioning care, governed by local public institutions and financed by federal funding. Research indicates that community health centers, writ large, deliver equally high-quality care, often better and more equitable care than other practices because of their emphasis on team-based and holistic care that addresses nonclinical wraparound services.^22^ Public entity health centers, as a subset, appear to perform just as well as traditional health centers, even as they see a higher proportion of uninsured patients.^23^

Public health centers provide an avenue for local public investment to support health centers, which generally rely on federal funds and philanthropy. They enable tight integration with traditional public health functions of a county, as well as attractive compensation and worker protections. Yet the expansion of public health centers is constrained by the 5% cap set by congress. To build out this “public–public” delivery model, policymakers could lift the 5% cap and enhance grant funding public instiuttions at the local level.

### Pharmaceuticals

Despite hosting the world's most robust and profitable private pharmaceutical sector, the United States also provides numerous essential medicines in the public sector. For over 125 years, state-owned and operated MassBiologics has developed, produced, and distributed immunotherapies, vaccines, monoclonal antibodies, and other biologics for free to MA residents.^24^ In the 1990s, the California Department of Public Health developed a treatment for infant botulism that it continues to manufacture and distribute at nonprofit rates today. The Walter Reed Pilot Bioproduction Facility produces vaccines and other biologics. These public institutions have historically focused on public health-related products, like vaccines, that are often underfunded by private sector pharmaceutical companies.^25,26^ They can assure resilient supply chains and offer medicines at or below cost.

The subsequent privatization of some of these facilities serves as a cautionary tale. Michigan produced vaccines and other biologics and distributed them for free to residents and at-cost to other public and nonprofit payers.^27,28^ During a national shortage of the tetanus-diphtheria vaccine in the 1990s, Michigan and Massachusetts were the only states not affected due to their in-house production capabilities.^29^ When the Michigan lab was privatized in 1998, it was the only US-based producer of anthrax^30^ and rabies vaccines.^31^ The lab's new private owners struggled to produce the anthrax vaccine,^32^ failing to secure Food and Drug Administration certification for its manufacturing facility from 1998 to 2002 and leaving the country without a domestic producer of this critical vaccine during a crucial moment given the 2001 anthrax attacks.^33^

Today, there are dire shortages in the generic drug market,^34,35^ and policymakers are beginning to look to the public sector in response. California announced plans to bring low-cost insulins and other drugs to market as early as next year, and several other states are considering following suit. Federally, there are proposals both for large-scale generics production and public R&D coupled with public-interest provisions on all resulting intellectual property. These public-option proposals deserve serious consideration, creating resilience and redundancy in our drug supply chains and strong manufacturing jobs for the future.

## Future policy directions

These examples illustrate that public institutions can deliver high-quality, efficient, and equitable care and produce vital medications. Public provisioning allows for local control of a community's care infrastructure, with the potential to tailor to local needs and serve as economic anchors.^36^ Discouraged from financializing or divesting from local economies, these institutions can integrate with public health initiatives and localized procurement strategies.^37^ Moreover, as health care is the largest workforce in the American economy, public provisioning can offer strong unionized jobs,^38^ often held by underrepresented groups.^39^ It should be viewed as a prolabor “public option” that will bolster corresponding worker power in the private sector.

Yet current policy limits the growth of public provisioning, despite its potential to meet urgent care capacity needs and drug shortages across demographic and socioeconomic strata. Policymakers could begin to address this by lifting the cap on public entity FQHCs and expanding the reach of health centers, generally, by increasing grant funding and broadening qualifying regions for health centers. A “new Hill–Burton”^40^ could emphasize investment through public hospitals and health districts. Similarly, state and federal investment dollars could be channeled to build pharmaceutical capacities, particularly in areas vulnerable to supply chain disruptions or generics and biosimilars lacking private investment. Ultimately, a new health care industrial policy must prioritize a rational production and allocation of capacity—and public provisioning can ensure that these public investments deliver a return for patients, the health care workforce, and their local communities.

## Supplementary Material

qxaf013_Supplementary_Data
